# Talent identification of 12-year old male Australian rules footballers: Physical advantages and prognosis for junior and senior national-level selection

**DOI:** 10.1371/journal.pone.0317336

**Published:** 2025-02-10

**Authors:** Paul Larkin, Gyan Wijekulasuriya, Sam Greer

**Affiliations:** 1 Institute for Health and Sport, Victoria University, Melbourne, Australia; 2 MSA Research Centre, Maribyrnong Sports Academy, Melbourne, Australia; Mugla Sitki Kocman University: Mugla Sitki Kocman Universitesi, TÜRKIYE

## Abstract

Talent identification focuses on the ability to identify and select young athletes who show potential for future sporting success. The aim of this study was to compare the anthropometry and physical performance of male youth Australian Football players selected and not selected into a high performance sports academy program. Its secondary aim was to determine whether selection into a talent development environment at 12 years old affects the odds of selection into subsequent junior national level representative and senior professional Australian Football programs. 168 youth males (11.7 ± 0.4 years) who nominated for the selection process to attend a specialised high school sports academy completed a series of physical and anthropometric assessments. The data collection period occurred over seven years (2013–2019), with a prognostic period between one to ten years. Results found selected 12 years olds were taller and had greater lower body power, speed and aerobic fitness than non-selected athletes. A combination of height, aerobic fitness, and lower body power best distinguished between selected and non-selected players. This discriminant analysis had high accuracy and greater sensitivity than specificity. Further, athletes selected into the talent development environment had a much greater likelihood of being selected into junior state/national (54-fold) and senior professional (5-fold) teams than non-selected athletes. Overall, the findings demonstrate some support for the prognostic ability of selection into a talent development program at 12 years old to predict later selection in the talent pathway. These findings support the role of talent development programs in the development of male Australian footballers.

## Introduction

Talent identification in sport focuses on the ability to identify and select young athletes who show potential for future success [1]. As a process, talent identification is recognising and interpreting current levels of performance to inform predictions relating to who has the best potential for future success as an elite adult athlete [1–3]. Thus, experienced talent identifiers (i.e., scouts, coaches) interpret current performance levels and attempt to predict future domain-specific potential. Consequently, the overarching aim of the talent identification process is to identify and select athletes who, in the future, will outperform athletes who are not identified or selected for talent development programs [1].

An issue with talent identification and development programs is the assumption factors which contribute to successful senior performance can be generalized and measured within a youth group to predict future ability [4,5]. Within an Australian Football context, the final stage of talent identification, selection by a professional team, is facilitated through the National Draft Combine, whereby talent identified youth players (i.e., 18 years of age) are assessed on a range of anthropometrical, physical, and technical skill assessments [6]. To determine the utility of the National Draft Combine to inform talent identification decisions, researchers have found strong associations between the performance on a range of National Draft Combine assessments, and selection into the elite Australian Football League competition [6–11]. These findings further demonstrate the importance of these attributes for elite level performance, whereby physical capacities (i.e., aerobic capacity, speed and power) have been found to distinguish between elite Australian Football team selection and non-selection, career progression and playing performance [12,13]. While these findings highlight some of the factors which may assist in the identification of talent at the final stage of the Australian Football talent identification process (i.e., selection into an elite senior competition; 18 years of age), there is still limited knowledge about the factors which may influence talent identification decisions early in the talent identification and development process (i.e., 12 years of age).

A key consideration within the talent identification process is the degree to which physical and anthropometric attributes affect selection during the initial stage of the talent identification process [14]. The initial stage of the talent identification process for Australian footballers occurs when an athlete is between 11 and 13 years old [15,16]. At this age athletes can be selected into club academies [16], state representative teams or school-based sports academies [17]. To our knowledge, while there are findings at the later stages of the talent identification process (i.e., National Draft Combine research; [6–11]), there is no empirical research which has examined the physical and anthropometric attributes which may impact early talent identification processes. Consequently, there is limited understanding of the predictive ability of physical and anthropometric attributes on selection during childhood/early adolescence in Australian Football and the subsequent impact on talent identification during later stages. Researchers have attempted to address this question within a soccer context, whereby the prognostic ability of performance tests conducted at a youth level to predict future adult performance has been assessed [14,18,19]. It should be noted that there are inconsistent findings within the literature, with some studies reporting positive associations with later playing performance [20–22], whereas others have found limited predictive ability of assessments [23–25].

A further consideration is the potential effect of talent identification biases, such as the relative age effect, on early-stage talent identification. The relative age effect is a consequence of age grouping within sport and describes how athletes born within the first quartile of an age group are talent identified at a higher rate compared to athletes born later in the year [26,27]. This likely occurs due to the larger amount of practice accumulated by relatively older athletes [26] and subsequent development of technical and tactical skills within this cohort [26,28]. Moreover, the relative age effect may affect talent development as relatively younger athletes are less likely to participate in mid- to late-stage talent development programs and be selected into elite sport [27]. The relative age effect has been observed within early-[16], mid-[16,28,29] and late-stage [16,29] male Australian football talent identification programs. Assessment of the relative age effect has occurred rarely during early-stage talent identification and whether it is present when selecting talent for a school-based sports academy is unknown.

While researchers have suggested performance tests have some prognostic value and assist in identifying young athletes who possess physical qualities associated with success in a sport, there is limited knowledge of this within an Australian Football context. Therefore, the purpose of this study was to identify the anthropometric (i.e., body height, body mass) and physical performance (i.e., countermovement jump; 5 m, 10 m and 20 m sprint) differences between youth Australian Football players selected or not selected into a high performance sports academy program. A further aim of this study was to determine the prognostic ability of selection into the academy program to predict selection into state and national representative Australian Football programs.

## Method

### Participants

In total, 168 youth males (Age =  11.7 ±  0.4 y, height =  150.7 ±  7.0 cm, body mass =  41.6 ±  8.1 kg) participated in the study. All participants had nominated for the selection process to attend a specialised high school sports academy the following year. As part of the selection process, participants completed a series of physical and anthropometric assessments. The data collection period occurred over a seven year period (10/05/2013–31/05/2019), however, it should be noted no data was collected in 2014. On average, 28 participants nominated for a trial each year, with a panel of four experienced youth Australian Football talent development coaches making selection decisions based on the participant’s performance. Approximately eight participants were selected each year, with a total of 48 participants being offered a position into the specialised high school sports academy over the seven year period. Ethical approval for the project was granted by the Victoria University Human Research Ethics Committee (HRE 23-024, Approved: 25-06-2024). Prior to participants attending the testing sessions, participant’s parents provided written informed consent for the recording and scientific use of data collected.

### Measures

All measurements were recorded in accordance with the procedures outlined by Larkin and colleagues [17].

#### Relative age.

The birthdates for each participant were self-reported. Then, the birthdate of each participant was separated into quartiles, using 1^st^ January of the birth year of each athlete cohort (2001-2008) as the cut-off date. Therefore, the following birth quartiles were used for assessments: Q1: January–March Q2: April–June; Q3: July–September; and Q4: October–December.

#### Standing height.

Height was measured using a Stadiometer with a sliding head piece in the Frankfort plane using the stretch stature method. Participant removed their shoes and socks and stood with feet together and the heels, buttocks, and upper part of the back touching the scale. The recorded measurement was taken at the end of a deep inward breath. All heights were recorded in centimetres (cm, TEM =  1.0 cm; [10]).

#### Body mass.

A calibrated electronic scale was used to measure body mass. The scales were placed on a hard floor surface (i.e., basketball court). Participants were asked to stand in the centre of the scale, with shoes and socks removed, with their weight evenly distributed on both feet while looking directly ahead. The measurement was recorded when the value stabilized. All body mass measurements were recorded in kilograms (kg), with a typical error of 1.0 kg [10].

#### Vertical jump.

Participant’s vertical jump was measured using a Vertec jump device (Swift Performance Equipment, Lismore, Australia). To measure vertical jump, participants’ standing reach was recorded first, by the participant standing side-on to the Vertec, and while keeping their heels on the floor, reaching upward with their dominant hand as high as possible, and displacing as many vanes as possible. The number of vanes displaced was recorded. Following this, participants were instructed to stand close to the Vertec for their jump. Take-off was from two feet, with an arm swing and countermovement used to jump as high as possible. At the height of the jump, participants displaced as many vanes as possible. Three stationary bilateral countermovement jumps were completed, with the highest vane displaced over the three jumps recorded. The difference between the maximum panes displaced and standing reach was recorded as the vertical jump height in centimeters (TEM =  2.0 cm; [30]).

#### Sprint performance.

Sprint times were recorded using electronic timing gates (Swift Performance Equipment, Lismore, Australia). A 20 m straight line course was set out on a basketball court, with the timing gates positioned at the start line (0 m) and 20 m line. Two cones were positioned at the 25 m mark as a run-off area. Participants were instructed to position themselves with their front foot touching the start line, in a crouched standing position with their body mass over their front foot, shoulders and hips square, and their back heel up. Participants were informed not to rock prior to the start, and a 3-point start position (i.e., one hand touching the ground) was not allowed. Participants were able to start when they were ready, eliminating reaction time, and instructed to sprint as fast as possible to the cones at the 25 m mark to ensure they did not decelerate before they reached the 20 m timing gates. Participants completed three maximal sprints, with a 2-minute recovery between sprints. The best time was recorded in seconds (sec), with a typical error of measurement of 0.03 seconds [30].

#### Multi-stage fitness test (MSFT).

Standard procedures were adopted for the MSFT. Participants were required to run back and forth between two marked lines spaced 20 m apart, within a designated time period. The time period decreased with each level, with the running speed standardized by prerecorded auditory cues (i.e., beeps) played through a portable speaker. The speaker was located in the center of the running area and positioned such that it would not interfere with the participants. The test was terminated when a participant was unable to reach the marked line twice in a row in accordance with the auditory cues, or by voluntary stopping. The recorded measure for the test was the final stage the participant was able to achieve before stopping. As the stage number is not a continuous variable, the level achieved was converted to a continuous variable for analysis purposes.

### Procedures

Over the seven year period, testing was conducted by tertiary educated strength and conditioning coaches employed by the specialized high school sports academy. During this time, each of the coaches conducted three to seven testing sessions. For all testing sessions, the same equipment was used, with testing occurring in the same indoor venue with similar environmental conditions at the same time period each year (May). Prior to testing all electronic equipment was calibrated.

Data collection was conducted in one session, with anthropometric measures (i.e., height and body mass) recorded first. All participants then completed a standardised 10-minute warm up with a qualified strength and conditioning coach. Based on previous recommendations, the physical tests were sequenced with the vertical jump completed first, followed by the 20m sprint, and finishing with the MSFT [30]. Appropriate rest was provided between each test. All data was obtained 26-06-2024 and collated onto a Microsoft Excel spreadsheet.

The physical and anthropometric data served as predictors for the participant’s selection into the high school academy. Then, selection into the academy was used as a predictor for later Australian Football success, which was defined as being selected for an independent state talent identification program from the age of 15 years (junior national level; School Sport Victoria U15s, U16s/U18s National Championships) or obtaining a professional contract in the Australian Football League (senior national level). As a result, the prognostic period was between five to ten years. To obtain information in relation to selection into a junior state talent identification program, all records of state team selections was obtained and reviewed for the prognostic period up until May 2024. Further, an individual search of each participant was conducted to cross check state representation results. Australian Football League level attainment was verified through an individual internet search of each participant to check level of senior football representation.

### Statistical analysis

Descriptive statistics were used to summarise the data, with the mean, standard deviation (SD), minimum, and maximum reported for all measures (i.e., standing height; body mass; vertical jump; sprint performance; MSFT). A Welch’s t-test were used to determine between group differences in physical performance measures. Point estimates for mean difference in populations, alongside 95% confidence intervals were calculated and effect sizes were calculated using Cohen’s *d* [31].

Step-up logistic regression (glm package, R Studio v 4.1.2) was used to determine the discriminant ability of the measures for talent identification into a junior Australian Football academy at 12 years of age. Logit scatter plots were inspected visually and showed that all covariates were linear. Cook’s distance was used to assess for outliers and none were identified. The variance inflation factor for all variables were approximately 1 indicating no multicollinearity. Selection status was the binary dependent variable (selected, non-selected) and independent variables included in the regression model were anthropometric (height, weight), birth quartile and physical fitness measures (MSFT, 20 m sprint, vertical jump). Data were separated into testing (70%) and training (30%) sets. Data in the testing set were used when testing different models and the model accuracy was calculated by comparing the accuracy of prediction of the training dataset to the testing data set models. Initially, all variables within the testing dataset were included in the modelling. Variables were then removed until the Akaike Information Criterion (AIC) was minimized which indicated the model with the best goodness of fit. An ROC curve was used to determine the specificity and sensitivity of the final model. A c-statistic was calculated to determine the ability of the model to discriminate selected and non-selected athletes. Calibration of the model occurred through visual inspection of a calibration curve. Standardised beta (β) ±  standard error were calculated for comparison in effect between variables of different scales in the modelling.

The prognosis of selection into a talent development environment to predict medium term success (i.e., junior state team selection/senior professional team selection) was determined using odds ratios. R Studio version 4.1.2 was used for all statistical analysis (packages: rstatix, glm). Results were considered statistically significant when p <  0.05 and Sawilowsky’s rule of thumb ([32]; very small, d =  0.01; small, d =  0.20; medium, d =  0.50; large, d =  0.80; very large, d =  1.20; huge, d =  2.00) was used to interpreted the magnitude of standardised effects (Cohen’s *d*, standardised β).

## Results

Logistic regression showed birth quartiles had no effect on selection (Q1–Q2: p =  0.702, Q1–Q3: p =  0.504, Q1–Q4: p =  0.446). Therefore, relative age was excluded from further discriminant analyses.

Selected athletes into the sports academy were taller (p <  0.001, ES =  0.80, Table 1), had greater vertical jump (p <  0.001, ES =  0.97), multistage fitness test score (p <  0.001, ES =  1.13) and lower 20 m sprint time (p <  0.001, ES =  0.81) when compared with unselected athletes at 12 years of age.

**Table 1:** Descriptive statistics and t test results for anthropometric and physical performance measures of selected and non-selected 12 year old Australian footballers in a school-based sports academy. Significance was accepted as p <  0.05 and effect sizes were interpreted using Sawizlowsky’s rule of thumb.

	Selected			Non-Selected						
Variable	n	Mean	SD	n	Mean	SD	Mean Difference	p-value	Effect Size	
Height (cm)	48	154.8	7.2	120	149.4	6.4	5.4	<0.001	0.80	Large
Mass (kg)	48	43.4	6.5	120	41.6	9.3	1.8	0.164	0.22	Small
Vertical Jump (cm)	48	40.1	5.9	120	34.9	4.8	5.3	<0.001	0.97	Large
Multistage Fitness Test (stage)	42	9.9	1.4	113	8.4	1.4	1.6	<0.001	1.13	Large
20 m Sprint (sec)	48	3.62	0.17	120	3.76	0.18	−0.14	<0.001	0.81	Large

The regression model (Table 2) with the lowest AIC which predicted selection into the sports academy at 12 years old included height (p =  0.010, β =  0.89 ±  0.34), MSFT (p <  0.001, β =  1.50 ±  0.39) and vertical jump (p =  0.010, β =  0.95 ±  0.37).

**Table 2:** Logistic regression model results for selected and non-selected 12 year old male Australian footballers in a high performance sports academy. Point estimates in standard (estimate) and normalised units (standardised beta) are reported. Statistical significance occurred when p <  0.05.

Coefficient	Estimate	Standard Error	Standardised Beta	z-score	p-value
Intercept	−35.50	8.667	0.000	−4.096	< 0.001
Height	0.127	0.049	1.135	2.582	0.010
Multistage Fitness Test	0.976	0.253	2.653	3.852	< 0.001
Vertical Jump	0.171	0.066	1.187	2.579	0.010

**Fig 1:**
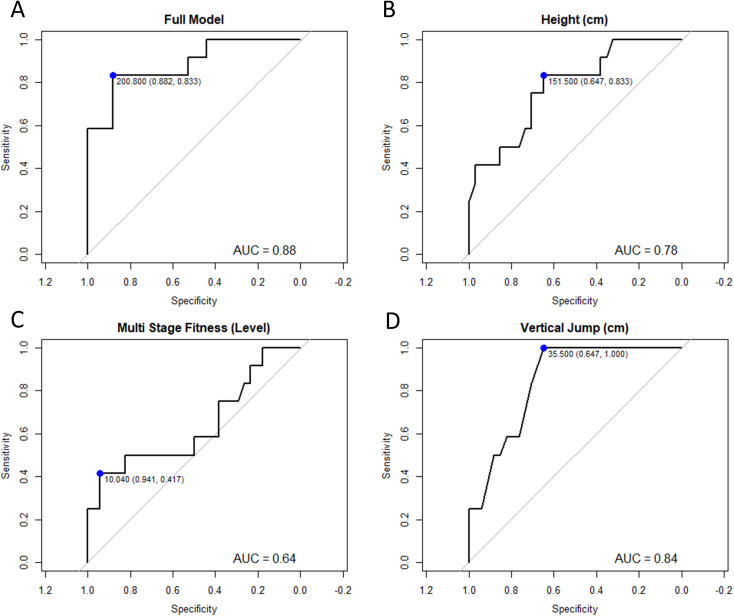
Receiver operator curves for A) full model, B) height, C) multistage fitness test and D) vertical jump height. Maximum likelihood estimate (blue dot) and area under the curve (AUC) for each curve and are displayed on each panel. Sensitivity and specificity of each curve (sensitivity, specificity) are displayed within the brackets alongside the maximum likelihood estimate.

Model accuracy was 84.8% and the c-statistic was 0.89. Visual inspection of the calibration curve showed no obvious outliers. The ROC curve (AUC: 0.88, Fig 1) showed that screening athletes based on the physical fitness maximum likelihood estimate exhibited high specificity (75%) and sensitivity (83%).

45% of selected athletes were subsequently selected in a junior (U15, U16, U18) state representative Australian football team and 4% were selected into the Australian Football League. 1% of non-selected athletes achieved junior state representative or Australian Football League selection. The odds of achieving junior national level was ~ 54 times greater for athletes selected in the sports academy (OR =  54.1) compared to athletes who attended selection trials and were not selected. The odds of achieving senior national level were ~ 5 times greater for athletes selected in the sports academy (OR =  5.4).

## Discussion

This study aimed to identify the anthropometric and physical performance factors associated with selection into an Australian Football sports academy program at 12 years of age and determine whether early-stage talent identification is prognostic for late-stage talent identification in the Australian Football pathway. This study showed that Australian footballers talent identified at 12 years old were taller and exhibited greater physical performance than non-identified athletes. Moreover, 12 year old selected footballers have higher odds of selection into junior state (School Sport Victoria U15s, U16s/U18s National Championships) and senior professional teams (Australian Football League) than non-selected footballers. Discriminant analysis showed that height, multistage fitness test score and vertical jump height were associated with selection at 12 years old with high accuracy, specificity and sensitivity.

Despite talent identification occurring at an early age (soccer: ~ 9 years old, Australian Football: ~ 12 years old) there have been a paucity of studies identifying the anthropometric and physical performance factors affecting selection at this age. This study is the first to report greater physical performance in selected compared to non-selected Australian footballers at ~ 12 years old. These are similar to the findings of earlier studies that showed physical performance discriminates talent identified and non-identified male Australian football athletes in U15 [33], U16 [29] and U18 [11,34] age groups. Aerobic fitness has been shown to discriminate selected and non-selected athletes across the timespan of the talent pathway [11,29,33,34] whilst lower body power and speed characteristics with discriminative power vary throughout the adolescent pathway [33–35] and for senior athletes [36].

A novel aspect of this study is the calculated odds of future selection in adolescent talent pathway and senior professional squads for a group of talent identified 12 year old Australian rules footballers. The results show that identifying talented Australian rules footballers at 12 years of age has prognostic ability for future junior and senior success. This is evidence that selection in a talent development environment at an early age is advantageous for future selection in the talent pathway. A previous study in German soccer showed that future professional footballers exhibited greater motor performance from U12 to U15 than footballers who did not attain a professional contract [37]. Our findings are somewhat in agreement as talent identified 12-year-old Australian rules footballers exhibited greater physical performance than non-talent identified players and had greater odds of talent selection throughout the talent pathway. Together, the evidence from these studies may be interpreted as evidence for the “innate abilities” hypothesis that underpins talent identification and development from a young age. Furthermore, Lehyr and colleagues [37] note that physical advantages during early adolescence are retained into middle adolescence and affect selection in the U15 age group and this study showed almost half (45%) of the athletes selected at 12 years old achieved state or national representative teams be-tween the ages of 15 and 18. Therefore, the selection decisions during early adolescence have reasonable prognosis for physical performance and Australian rules football team selection during late adolescence.

An alternate explanation for the results of the study is that they reflect a maturation selection bias which may affect the selection and retention of talent in Australian rules football from a young age. This is an issue in talent identification as biases in selection affect which players gain access to the talent development pathway and obscure talented athletes behind physical advantages [26,38]. Taller athletes at ~ 12 years old are typically early maturing [39,40] and early maturing athletes exhibit greater physical fitness compared to later maturing athletes during early adolescence [39,40]. However, there are no differences in physical performance between these groups once they reach adulthood [41]. Athletes excluded due to maturation selection bias have limited exposure to high quality coaching and can be systemically excluded from the talent pathway [39,40]. Another selection bias is the relative age effect [16,26,27], however there was no evidence of this bias in this study. Other studies have shown early maturers are preferentially selected to U13 to U15 [33,35], and U16 [42] Australian rules football talent pathway programs and a previous study has shown a relative age effect across the Australian football pathway, including U10–U12 age groups [16]. A major limitation of this study is that a measure of maturation status was not collected. Future studies of talent identification in early adolescent Australian rules footballers must include either the calculation of maturity offset [43] and/or percentage of predicted adult height [44] to determine whether maturation selection bias occurs during Australian rules football talent identification at 12 years old. Considering the likely systemic maturation selection bias within the male Australian football talent selection pathway, it may be useful to trial the effectiveness of talent selection strategies such as bio-banding [39] in Australian rules football to overcome these biases.

A potential flow on effect of maturation selection bias and talent identification at an early age is early specialisation and dropout from sport. Early specialisation in sport is characterized as a singular sport high volume training program initiated at an early age [45]. Participation in a training environment with these markers is associated with high performance at an early chronological age but low performance during adulthood [46]. The substantially greater odds of junior (U15, U16, U18) state selection compared to senior AFL selection in this study may be evidence of an early specialisation effect in Australian rules football. Recent reviews suggest that junior elite performance and senior elite performance have low to no correlation [46] and are developed in divergent talent environments [47]. Descriptions of talent development environments in the Australian football pathway are rare and the efficacy of these environments have not been reported to date. Future research should evaluate the efficacy of different talent development environments on physical, technical, tactical and psychological development which may enable a “best practice model” to be developed for talent development in junior male Australian football athletes.

Other limitations of this study include that it was undertaken within a single talent development environment. Athletes selected to this environment had greater odds of selection in future talent pathway teams, however these findings may not be transferrable to other talent development environments. Therefore, a multi-site evaluation of the factors affecting senior professional selection in the Australian football talent pathway would be valuable to evaluate the efficacy of the Australian rules football talent development system. Moreover, multiple studies have shown that assessing multiple dimensions of skill improves the discriminatory power of testing batteries used for talent identification [29,33,48]. Therefore, whilst this study found that physical factors have high discriminatory power at 12 years of age, talent development and assessment at this age should encompass a multi-dimensional approach and future studies should determine the discriminatory power of a multi-dimensional assessment battery in young Australian rules footballers.

## Conclusion

This study showed that physical performance discriminates selected and non-selected 12 year old male Australian footballers into a high performance sports academy program. Furthermore, there is some support for the prognostic ability of talent selection at 12 years of age to predict selection into state and national representative Australian Football programs. This is demonstrated by selection into a high performance sports academy program increasing the odds of achieving junior or senior national level success, compared to non-selection.
